# Supplementary material for: p32-Dependent p38 MAPK Activation by Arginase II Downregulation Contributes to Endothelial Nitric Oxide Synthase Activation in HUVECs
Source: Cells. 2020 Feb 8;9(2):392. doi: 10.3390/cells9020392 (PMC7072651; doi:10.3390/cells9020392)
Supplement: Supplementary file 1 [file cells-09-00392-s001.zip › cells-682922-supplementary.pptx]

## Slide 1
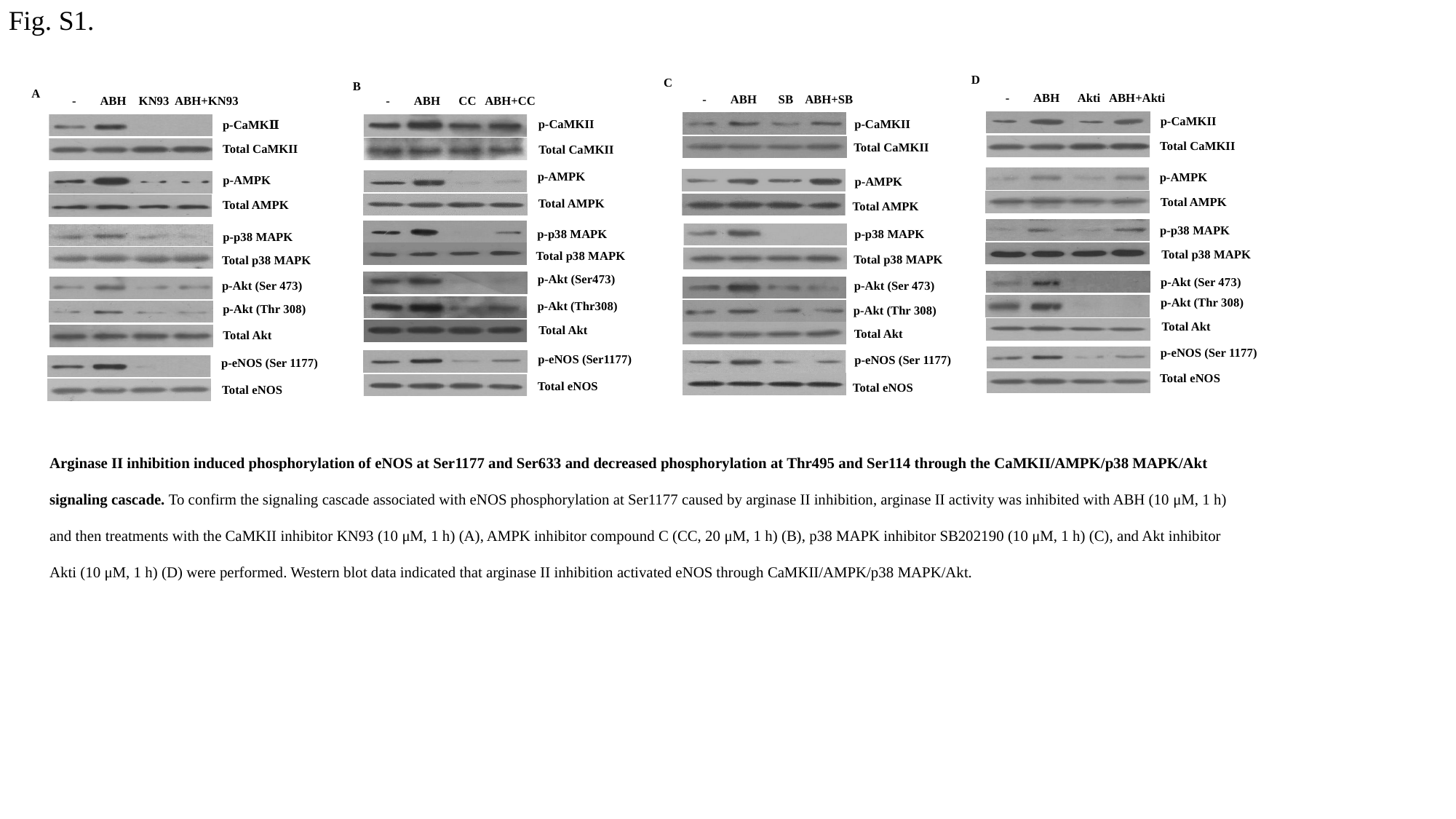

Fig. S1.
D
C
B
A
- ABH Akti ABH+Akti
- ABH SB ABH+SB
- ABH KN93 ABH+KN93
- ABH CC ABH+CC
p-CaMKII
p-CaMKII
p-CaMKII
p-CaMKⅡ
Total CaMKII
Total CaMKII
Total CaMKII
Total CaMKII
p-AMPK
p-AMPK
p-AMPK
p-AMPK
Total AMPK
Total AMPK
Total AMPK
Total AMPK
p-p38 MAPK
p-p38 MAPK
p-p38 MAPK
p-p38 MAPK
Total p38 MAPK
Total p38 MAPK
Total p38 MAPK
Total p38 MAPK
p-Akt (Ser473)
p-Akt (Ser 473)
p-Akt (Ser 473)
p-Akt (Ser 473)
p-Akt (Thr 308)
p-Akt (Thr308)
p-Akt (Thr 308)
p-Akt (Thr 308)
Total Akt
Total Akt
Total Akt
Total Akt
p-eNOS (Ser 1177)
p-eNOS (Ser1177)
p-eNOS (Ser 1177)
p-eNOS (Ser 1177)
Total eNOS
Total eNOS
Total eNOS
Total eNOS
Arginase II inhibition induced phosphorylation of eNOS at Ser1177 and Ser633 and decreased phosphorylation at Thr495 and Ser114 through the CaMKII/AMPK/p38 MAPK/Akt signaling cascade. To confirm the signaling cascade associated with eNOS phosphorylation at Ser1177 caused by arginase II inhibition, arginase II activity was inhibited with ABH (10 μM, 1 h) and then treatments with the CaMKII inhibitor KN93 (10 μM, 1 h) (A), AMPK inhibitor compound C (CC, 20 μM, 1 h) (B), p38 MAPK inhibitor SB202190 (10 μM, 1 h) (C), and Akt inhibitor Akti (10 μM, 1 h) (D) were performed. Western blot data indicated that arginase II inhibition activated eNOS through CaMKII/AMPK/p38 MAPK/Akt.

## Slide 2
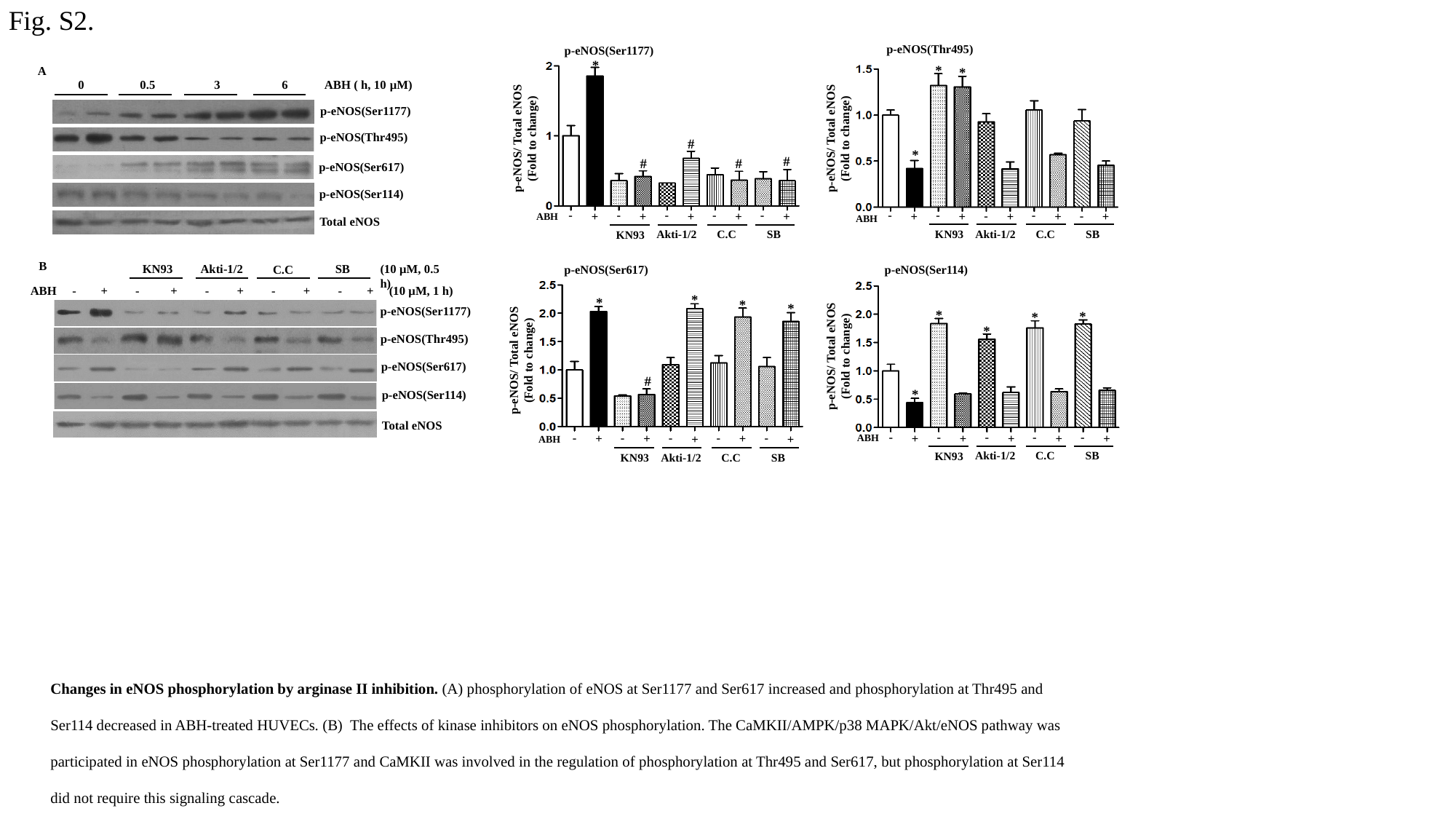

Fig. S2.
p-eNOS(Thr495)
p-eNOS(Ser1177)
*
*
A
*
0 0.5 3 6 ABH ( h, 10 μM)
*
p-eNOS(Ser1177)
p-eNOS/ Total eNOS
(Fold to change)
p-eNOS/ Total eNOS
(Fold to change)
p-eNOS(Thr495)
#
*
#
#
#
p-eNOS(Ser617)
p-eNOS(Ser114)
-
-
-
-
-
-
-
-
-
-
+
+
+
+
+
+
+
+
+
+
ABH
ABH
Total eNOS
SB
C.C
Akti-1/2
KN93
SB
C.C
Akti-1/2
KN93
B
(10 μM, 0.5 h)
Akti-1/2
SB
KN93
C.C
ABH - + - + - + - + - + (10 μM, 1 h)
p-eNOS(Ser1177)
p-eNOS(Thr495)
p-eNOS(Ser617)
p-eNOS(Ser114)
Total eNOS
p-eNOS(Ser617)
p-eNOS(Ser114)
*
*
*
*
*
*
*
*
p-eNOS/ Total eNOS
(Fold to change)
p-eNOS/ Total eNOS
(Fold to change)
#
*
-
-
-
-
-
-
-
-
-
-
+
+
+
+
+
+
+
+
+
+
ABH
ABH
SB
C.C
Akti-1/2
KN93
SB
C.C
Akti-1/2
KN93
Changes in eNOS phosphorylation by arginase II inhibition. (A) phosphorylation of eNOS at Ser1177 and Ser617 increased and phosphorylation at Thr495 and Ser114 decreased in ABH-treated HUVECs. (B) The effects of kinase inhibitors on eNOS phosphorylation. The CaMKII/AMPK/p38 MAPK/Akt/eNOS pathway was participated in eNOS phosphorylation at Ser1177 and CaMKII was involved in the regulation of phosphorylation at Thr495 and Ser617, but phosphorylation at Ser114 did not require this signaling cascade.

## Slide 3
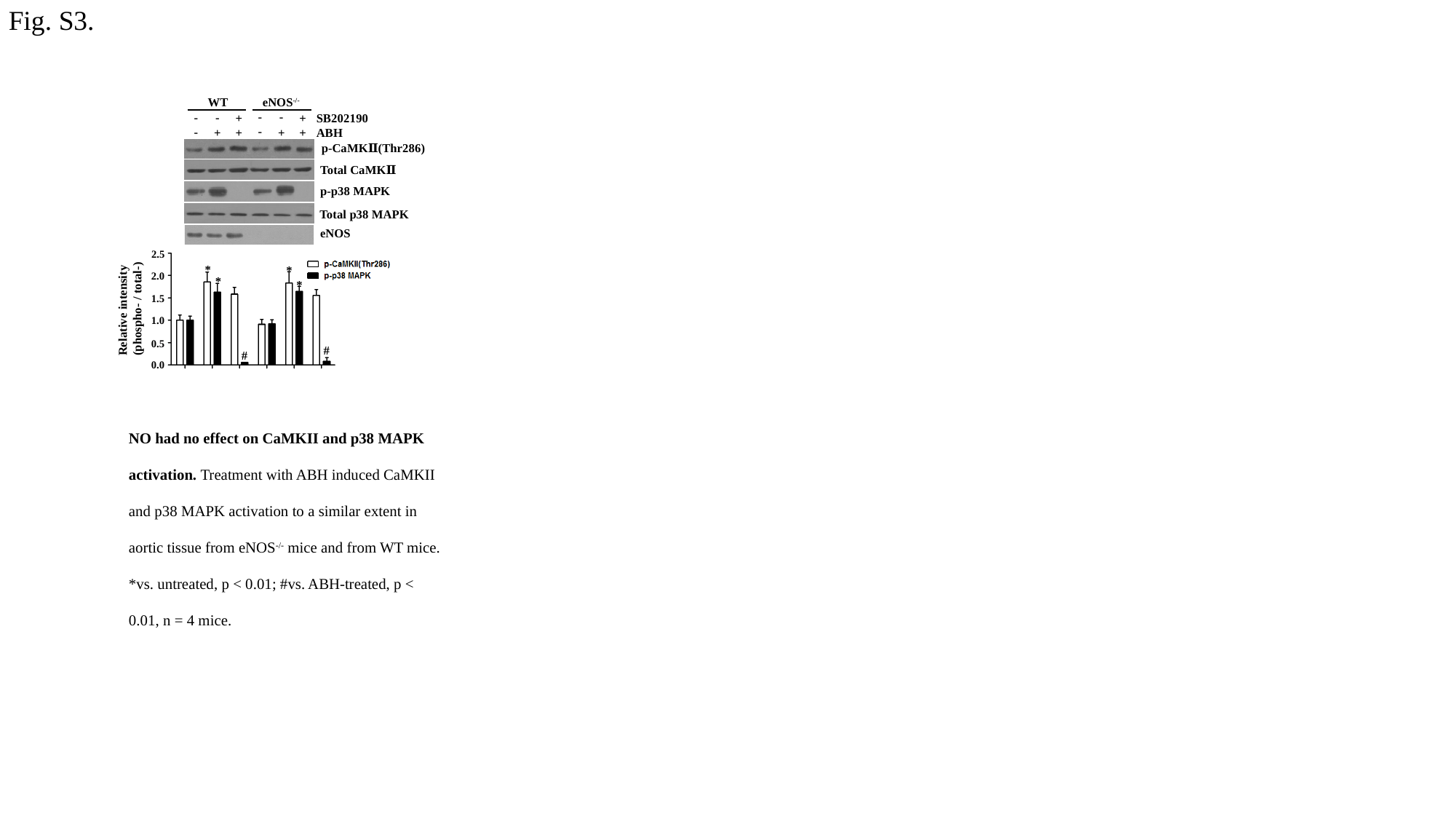

Fig. S3.
WT
eNOS-/-
-
-
-
-
-
-
SB202190
ABH
+
+
+
+
+
+
p-CaMKⅡ(Thr286)
Total CaMKⅡ
p-p38 MAPK
Total p38 MAPK
eNOS
2.5
*
*
2.0
*
*
1.5
Relative intensity
(phospho- / total-)
1.0
0.5
#
#
0.0
NO had no effect on CaMKII and p38 MAPK activation. Treatment with ABH induced CaMKII and p38 MAPK activation to a similar extent in aortic tissue from eNOS-/- mice and from WT mice. *vs. untreated, p < 0.01; #vs. ABH-treated, p < 0.01, n = 4 mice.

## Slide 4
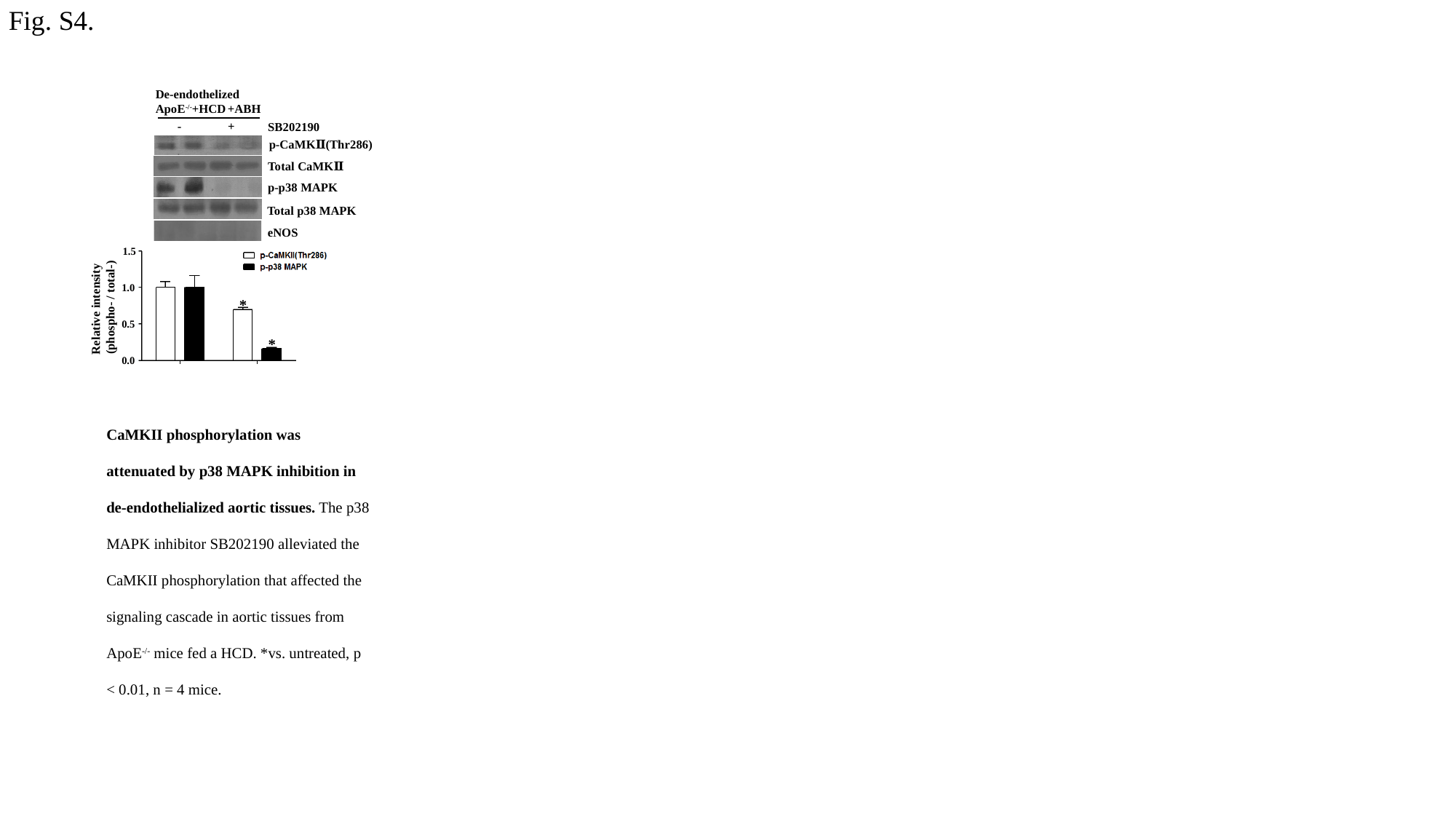

Fig. S4.
De-endothelized
ApoE-/-+HCD +ABH
- +
SB202190
p-CaMKⅡ(Thr286)
Total CaMKⅡ
p-p38 MAPK
Total p38 MAPK
eNOS
1.5
1.0
Relative intensity
(phospho- / total-)
*
0.5
*
0.0
CaMKII phosphorylation was attenuated by p38 MAPK inhibition in de-endothelialized aortic tissues. The p38 MAPK inhibitor SB202190 alleviated the CaMKII phosphorylation that affected the signaling cascade in aortic tissues from ApoE-/- mice fed a HCD. *vs. untreated, p < 0.01, n = 4 mice.

## Slide 5
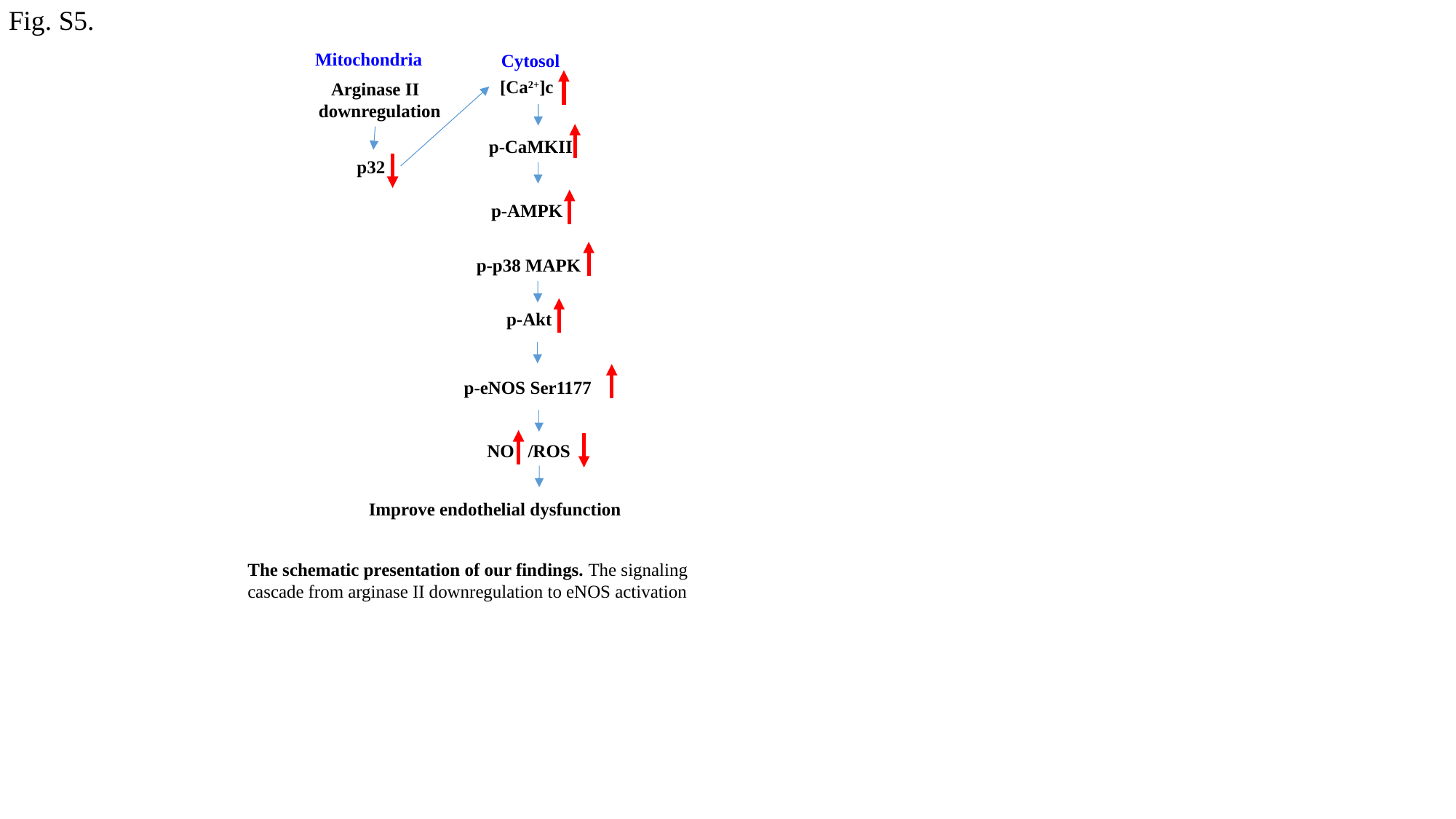

Fig. S5.
Mitochondria
Cytosol
[Ca2+]c
Arginase II
 downregulation
p-CaMKII
p32
p-AMPK
p-p38 MAPK
p-Akt
p-eNOS Ser1177
NO /ROS
Improve endothelial dysfunction
The schematic presentation of our findings. The signaling cascade from arginase II downregulation to eNOS activation
